# Plant Cell Cultures as a Tool to Study Programmed Cell Death

**DOI:** 10.3390/ijms22042166

**Published:** 2021-02-22

**Authors:** Massimo Malerba, Raffaella Cerana

**Affiliations:** 1 Dipartimento di Biotecnologie e Bioscienze, Università degli Studi di Milano-Bicocca, 20126 Milan, Italy; massimo.malerba@unimib.it; 2 Dipartimento di Scienze dell’Ambiente e della Terra, Università degli Studi di Milano-Bicocca, 20126 Milan, Italy

**Keywords:** cell cultures, programmed cell death, reactive oxygen species, reactive nitrogen species

## Abstract

Programmed cell death (PCD) is a genetically controlled suicide process present in all living beings with the scope of eliminating cells unnecessary or detrimental for the proper development of the organism. In plants, PCD plays a pivotal role in many developmental processes such as sex determination, senescence, and aerenchyma formation and is involved in the defense responses against abiotic and biotic stresses. Thus, its study is a main goal for plant scientists. However, since PCD often occurs in a small group of inaccessible cells buried in a bulk of surrounding uninvolved cells, its study in whole plant or complex tissues is very difficult. Due to their uniformity, accessibility, and reproducibility of application of stress conditions, cultured cells appear a useful tool to investigate the different aspects of plant PCD. In this review, we summarize how plant cell cultures can be utilized to clarify the plant PCD process.

## 1. Introduction

Programmed cell death (PCD) is a genetically controlled suicide process present in all living beings with the scope of eliminating cells unnecessary or detrimental for the proper development of the organism. PCD plays a pivotal role in the plant lifestyle and it is involved in several developmental (senescence, formation of tracheary elements, sex determination, aerenchyma formation, endosperm and aleuron maturation) and pathological contexts (response to stresses and to pathogen attack) [1,2]. Thus, its study is a main goal for plant scientists. PCD process is organized in three phases. The first one is the induction phase, where the cells receive a wide range of extra- or intracellular signals (developmental input, pathogen attack, signals from neighboring cells, abiotic or biotic stresses). The second one is the effector phase, where the signals are elaborated to activate the death machinery. The third one is the degradation phase, where the activity of the death machinery causes the controlled destructuring of fundamental cell components [3]. The degradation phase shows a set of hallmarks that can be used to identify cells undergoing PCD. These hallmarks include shrinkage of cellular and nuclear membrane, activation of specific cysteine proteases called caspases, and activation of specific endonucleases able to cleave DNA in controlled fragments (laddering) [3]. Unlike animals, where well-described forms of PCD (for example apoptosis) are reported, in plants, the PCD process is still poorly understood and the term PCD is widely used to describe cell death observed in different tissues and organs. At present, in plants, at least three forms of PCD have been described and cataloged on the basis of both cellular morphology and the main cellular compartment involved in the process. The “nucleus first form” is observable during the hypersensitive response to pathogen attack and it is similar to animal apoptosis for the presence of specific hallmarks, involvement of mitochondria included. The “chloroplast first form” is observable during foliar senescence, while the “vacuole first form” is observable during the maturation of vascular elements and during aerenchyma formation [4].

## 2. Main Advantages of Studying PCD in Plant Cell Cultures

The in-depth study of the mechanism of plant PCD can often be a hard work as this process generally occurs in a small group of cells surrounded by the large number of uninvolved cells present in whole plant and in complex tissues. For a number of reasons, cultured cells appeared an attractive model to several plant biologists to face this matter. For example, large quantities of cell material can be easily maintained or quickly generated from stocks to furnish investigators with a great number of fast-dividing and relatively homogeneous cells. In addition, the greater accessibility makes cell cultures a good system to which different compounds or stress conditions can be easily furnished to or removed from. Finally, with vital dyes such as Evans Blue or fluorescein diacetate, it is relatively simple to follow the growth or death response of cells to different stressors by removing them at time intervals from the culture batches or by observing the fate of single cells in real time. To further characterize the PCD process, it is relatively easy to assess under the microscope any visible morphological changes that occur in cultured cells induced to undergo PCD [5]. For example, nucleus condensation, a modification often occurring during PCD, is easily observable with 4′,6′-diamidino-2-phenylindole (DAPI). Thus, a large set of cell cultures, in particular (but not only) those obtained from the model plants *Arabidopsis thaliana* and *Nicotiana tabacum*, has been proposed and utilized to investigate plant PCD [5]. After some early indications ([6] and references therein), in the last years, several different conditions have been proven able to induce PCD in cell cultures. In this review, we summarize the literature on PCD induced by biotic and abiotic stresses useful to clarify the process in plants.

## 3. PCD Induced in Cell Cultures by Biotic Stress

Several toxins and metabolic products obtained by microorganisms and fungi can induce PCD in cell cultures, as summarized in Table 1.

For example, in *Acer pseupdoplatanus* L.-cultured cells, tunicamycin, an inhibitor of N-linked protein glycosylation produced by *Streptomyces lysosuperificus*, and brefeldin A, an inhibitor of protein trafficking from the Golgi apparatus produced by *Eupenicillium brefeldianum*, induce a PCD with apoptotic features such as reactive oxygen species (ROS) accumulation, changes in cell and nucleus morphology, and specific DNA fragmentation [7]. In the same experimental material, fusicoccin a well-known activator of the plasma membrane H^+^-ATPase produced by *Phomopsis amygdali* induces PCD with similar characteristics [8]. The well-identified target of these molecules permitted to test the role of specific cell compartments or physiological functions in the induction, development, and execution of plant PCD process. In particular, investigation conducted with fusicoccin showed that the phytotoxin-induced PCD involves changes in actin cytoskeleton [24] and utilizes the plant hormone ethylene as regulative molecule in addition to ROS and reactive nitrogen species (RNS) [25]. Interestingly, inhibition of cytochrome *c* release from the mitochondrion by cyclosporin A markedly prevents the fusicoccin-induced PCD [26], and recently a possible role as signaling molecule for peroxynitrite has been proposed [27]. These results also sustain the fundamental role of cytochrome *c* and peroxynitrite in the induction of PCD process in plants. In *Arabidopsis thaliana* cultures, thaxtomin A, an inhibitor of cellulose biosynthesis produced by *Streptomyces scabiei*, induces a PCD dependent on active gene transcription and de novo protein synthesis and that displays apoptotic-like features such as specific DNA fragmentation [9]. Interestingly, addition of auxin to *Arabidopsis* cell cultures prevents thaxtomin-induced PCD possibly by stabilizing the plasma membrane–cell wall–cytoskeleton continuum [28]. In tobacco BY-2 (*Nicotiana tabacum* L. cv. Bright Yellow 2) cell suspensions metabolic products present in the *Alternaria alternata* culture filtrate induce a PCD dependent on ROS generation that shows cytoplasm shrinkage, chromatin condensation, and DNA laddering [10]. Interestingly, the PCD induced in tobacco BY-2 cells by *Pectobacterium carotovorum* and *Pectobacterium atrosepticum* is reduced by culture filtrate of non-pathogenic *Streptomyces* sp. OE7 that through cytosolic Ca^2+^ changes and generation of ROS induces defense responses [29]. This highlights the complexity of the interactions between microorganisms and plants and the need for further investigations. In tobacco cv. NC89-cultured cells, fusaric acid, a non-specific toxin produced mainly by *Fusarium* spp., causes PCD with mechanism that is not well understood that, however, involves ROS overproduction and mitochondrial dysfunction [11]. In fact, pre-treatment of tobacco cells with the antioxidant molecule ascorbic acid and with the nicotinamide adenine dinucleotide phosphate (NADPH) oxidase inhibitor diphenyl iodonium significantly reduces the fusaric acid-induced accumulation of dead cells as well as the increase in caspase-3-like protease activity. Moreover, oligomycin and cyclosporine A, inhibitors of the mitochondrial ATP synthase and the mitochondrial permeability transition pore, respectively, also reduce the rate of fusaric acid-induced cell death [11]. PCD induced in cell cycle-synchronized tobacco BY-2 cells by application of culture filtrates of *Erwinia carotovora* involves changes in vacuole shape and disassembly of endoplasmic actin filaments [12]. In tobacco BY-2 cultures, deoxynivalenol, a mycotoxin synthesized by *Fusarium culmorum* and *Fusarium graminearum*, induces a PCD sustained by different cross-linked pathways involving ROS generation linked, at least partly, to a mitochondrial dysfunction and to transcriptional downregulation of the alternative oxidase (Aox1) gene and showing regulation of ion channel activities participating in cell shrinkage [13]. Interestingly, this mycotoxin is also able to induce PCD in animal cells, but with different characteristics. This suggests the presence of different ways to induce PCD between animals and plants (original articles cited in [13]). Some metabolites able to induce PCD in plant cultured cells can originate from the degradation of cellular components or are produced by the primary and secondary metabolism of microorganisms and plants. For example, ceramides, lipids derived from the membranes of eukaryotic cells, can induce PCD in *Arabidopsis* cultures in a Ca^2+^-dependent manner. In fact, the calcium channel-blocker lanthanum chloride substantially reduces the amount of ceramide-induced cell death [14]. Interestingly, in the same material, sphingolipids can reduce apoptotic-like PCD induced by different treatments, ceramides and heat stress included [30]. Moreover, in tomato suspensions, cell death induced by camptothecin, fumonisin B1, and CdSO_4_ is regulated by phosphatidic acid. This cell death involves ROS and ethylene, depends on caspase-like proteases, and expresses morphological features of apoptotic-like PCD such as protoplast shrinkage and nucleus condensation [15]. Reactive carbonyl species (namely, acrolein, shown in Table 1) derived from lipid peroxidation can activate caspase-3-like proteases to initiate PCD in tobacco BY-2 cultures [16]. In the same experimental material, narciclasine (NCS), a plant growth inhibitor isolated from the secreted mucilage of *Narcissus tazetta* bulbs, can induce typical PCD-associated morphological and biochemical changes, namely, cell shrinkage, chromatin condensation, and nuclear DNA degradation [17]. Among primary and secondary metabolites, the triterpene saponins (namely, medicagenic acid, shown in Table 1) from alfalfa (*Medicago sativa*) applied to *Populus alba* cell cultures induce a PCD dependent on RNS and ROS production and showing changes in nucleus morphology and chromatin condensation [18]. In tobacco BY-2 cultures, juglone (5-hydroxy-1,4-naphthoquinone) causes cell death with ROS overproduction accompanied by formation of apoptic-like nuclear bodies (indication of DNA fragmentation) and DNA hypomethylation [19]. In *Vitis labrusca* suspension cultures, L-alanine is the only amino acid able to induce PCD accompanied by DNA fragmentation, expression of defense-related genes, and accumulation of phenolic compounds [20]. Plant phytoregulators can also activate PCD in plant cell cultures. For example, high levels of cytokinins (namely, 6-benzylaminopurine, shown in Table 1) induce PCD in *Arabidopsis* cultures by accelerating a senescence process characterized by DNA laddering and expression of specific senescence markers [21]. In the same material acetylsalicylic acid, a derivative from the plant hormone salicylic acid induces typical PCD-linked morphological and biochemical changes, namely, cell shrinkage, nuclear DNA degradation, loss of mitochondrial membrane potential, cytochrome *c* release from mitochondria, and induction of caspase-like activity [22]. Finally, in *Acer pseudoplatanus* cultures, chitosan, the non-toxic and inexpensive compound obtained by deacetylation of chitin, the main component of the exoskeleton of arthropods as well as of the cell walls of many fungi, induces a PCD mediated by ROS and RNS accumulation and showing changes in gene expression and specific DNA fragmentation [23].

## 4. PCD Induced in Cell Cultures by Abiotic Stress

Several abiotic stresses ranking from different chemicals such as heavy metals and dyes to ambient growth conditions can induce PCD in plant cell cultures, as summarized in Table 2.

For example, cadmium is a potent inducer of PCD in plants and in tobacco BY-2-cultured cells; this process involves alterations in cell and nucleus morphology and appearance of autophagic bodies [31]. In the same experimental material, aluminum oxide nanoparticles induce a PCD form closely connected to loss of mitochondrial potential, enhancement of caspase-like activity, and DNA fragmentation [32]. In *Viola tricolor* L.-cultured cells, zinc and lead ions stimulate a PCD form showing DNA fragmentation and activation of caspase-like and papain-like cysteine proteases [33]. Interestingly, the indoleamine melatonin protects tobacco BY-2-cultured cells from lead stress by inhibiting cytochrome *c* release, thereby preventing the activation of the cascade of processes leading to cell death [46]. Other important environmental pollutants able to induce PCD in cultured plant cells are aromatic compounds. In fact, fluoranthene causes DNA fragmentation and oxidative stress in tobacco BY-2 suspension cultures [34]. Rose Bengal dye in *Arabidopsis thaliana* cell suspension cultures requires functional chloroplasts to activate a PCD process showing ROS accumulation and specific gene activation [35], and the herbicide dinitro-*o*-cresol induces DNA fragmentation, activation of caspase-3-like proteins, and release of cytochrome *c* from mitochondria in soybean (*Glycine max*) suspension cell cultures [36]. Other chemicals able to induce PCD are 1-butanol, which in *Populus euphratica* cell cultures causes shrinkage of the cytoplasm, DNA fragmentation, condensed or stretched chromatin, and the activation of caspase-3-like proteases [37] and ATP, which when externally added to the same cell cultures causes elevation of cytosolic Ca^2+^ levels, ROS accumulation, and cytochrome *c* release [38]. As far as environmental conditions are concerned, heat stress (HS) is a potent inducer of PCD in plants, where it causes important yield losses. HS study in cultured cells has permitted to elucidate some aspects of its induction, thus helping in the reduction of losses. For example, in tobacco BY-2-cultured cells, HS induces PCD, showing apoptotic features such as cytoplasmic shrinkage, DNA fragmentation, ROS accumulation, activation of caspase-3-like proteases, and induction of defense-related genes [39,47]. Some of these effects of HS are prevented by selenium [47] and depend on peroxynitrite accumulation [48], thus sustaining the fundamental role of oxidative stress in the induction of HS-dependent PCD. This view is also sustained by the analysis of the soluble proteome of tobacco cells subjected to HS and by custom microarray analysis of gene expression during PCD of *Arabidopsis thaliana*-cultured cells. Both these molecular investigations show the induction of genes related to oxidative stress resistance [49,50]. Another environmental condition that is able to induce PCD is salinity. Interestingly, the comparison of the responses to salt stress of suspension-cultured cells from the halophyte *Cakile maritima* and the glycophyte *Arabidopsis thaliana* shows that both species present similar dysfunction of mitochondria and caspase-3-like activation but the salt-tolerant *C. maritima* can better resist to stress due to a higher ascorbate pool able to mitigate the oxidative stress generated in response to NaCl [40]. O_3_ exposure also induces PCD dependent on ROS generation in cell suspensions of *Arabidopsis thaliana* [41]. Light also seems to be an important environmental factor able to regulate PCD. Darkness enhances cell death but flavonoids and darkness lower PCD during senescence of *Vitis vinifera* cell suspensions [42], pointing out the complexity of PCD regulation in plants. In tobacco BY 2-cultured cells, UV-B overexposure induces a PCD form showing typical apoptotic morphological features such as cell shrinkage, condensation of chromatin in perinuclear areas, and formation of micronuclei [43]. The nutritional aspect is also important. In fact, simultaneous depletion of sugar and phosphate is associated with PCD, showing nuclear DNA degradation in suspension cultures of maritime pine (*Pinus pinaster* Ait.) [44].

Very interesting results have been obtained from experiments performed in a cell cycle-synchronized *Arabidopsis thaliana* cell suspension culture treated with four physiological stressors (polyethylene glycol, mannose, H_2_O_2_, ethylene) in the late G2 phase. In these cultures, depending on the cell death inducer, there are significant differences in the appearance of specific PCD hallmarks. In fact, polyethylene glycol, mannose, and H_2_O_2_ cause DNA fragmentation and cell permeability to vital stains, and produce corpse morphology corresponding to apoptotic-like PCD. Instead, ethylene (a plant hormone associated with senescence) causes permeability of cells to vital stains without concomitant nuclear DNA fragmentation and cytoplasmic retraction but with very high ROS production, leading to severe oxidative stress [45]. Similarly, in tobacco BY 2-cultured cells, zinc oxide nanoparticles cause cell death depending on oxidative stress and lipid peroxidation [51], and in grapevine suspension cell cultures, different concentrations of silver ions cause cell death with different characteristics [52]. Thus, depending on the genotype/species and level of stress, the same factors may cause different responses. Low stress levels permit the repair of cell damage, moderate stress levels may induce PCD, and uncontrollable stress levels potentially lead to accidental cell death (necrosis, see also Section 5). This is particularly evident with abiotic stressors such as heavy metals and externally added compounds such as plant hormones and H_2_O_2_ (original articles cited in [53]).

## 5. Future Perspectives and Conclusions

**Scheme 1:**
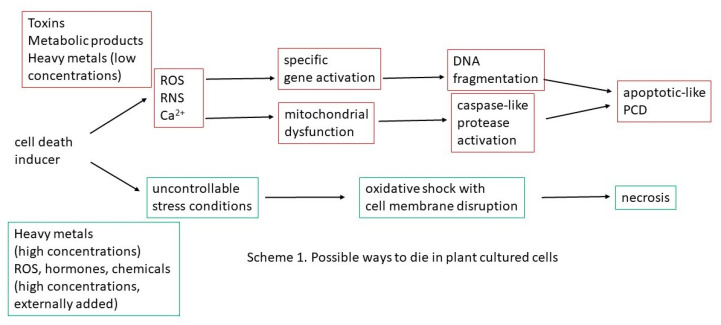
Possible ways to die in plant-cultured cells.

The use of cultured cells, a simple and controllable system valuable for physiological studies because it minimizes variability and facilitates the analysis of cellular features, permits the investigation of some aspects of the induction and progression process of plant PCD. In particular, the possibility to evaluate the effects of different inducers in a stable and reproducible manner made it possible to investigate the existence of multiple pathways to perform PCD in plants. For example, the comparison among the effect of tunicamycin, brefeldin A and fusicoccin and the lack of additivity when these inducers are furnished in combination to *Acer pseudoplatanus* cells, permitted to hypothesize for these inducers the existence of the same or largely coincident pathways involving mitochondria and endoplasmic reticulum to induce PCD [54]. On the other hand, the discrepancy between the number of dead cells and the number of cells showing specific DNA fragmentation suggests the presence of different types of PCD in these cultures [54]. This permits the hypothesis of the existence of at least two ways to die in cultured plant cells (Scheme 1): apoptotic-like PCD and necrosis.

The apoptotic-like PCD is probably the most present in cultured cells and occurs during developmental processes and in response to pathogen attack (Table 1 and Table 2). The biotic and abiotic cell death inducers stimulate ROS and RNS production and changes in cytosolic Ca^2+^ levels. These second messengers induce activation of specific genes, leading to DNA fragmentation as well as mitochondrial dysfunction with activation of caspase-like proteases, finally resulting in apoptotic-like PCD. The necrotic death involves swelling of the protoplast and loss of the integrity of the plasma membrane and occurs during the response to severe insults that cause an oxidative uncontrolled shock.

The absence of cellular differentiation in many suspension cultures is a main advantage when the basal responses of cells during the induction and execution of cell death programs are investigated. However, it is possible to obtain suspension cultures not totally undifferentiated to study some developmental processes. In fact, the developmental cell deaths that occur in culture are often more amenable to investigation than their in vivo counterparts. A classic example was the use of the xylogenic Zinnia (*Zinnia elegans*) cell culture as an efficient system for studies on xylogenesis, an example of the “vacuolar first” form of PCD. The developmental program of xylem differentiation in planta is well preserved in this in vitro experimental model. This allows for easy identification of signaling molecules and observations of the changes in the morphology of the cellular organelles [55]. The background provided by the xylogenesis research in Zinnia makes it possible to utilize a similar approach to study, in a simplified model, cell cultures other fundamental processes for the plant lifestyle such as embryogenesis, sex determination, or senescence.

In conclusion, the importance and complexity of the PCD process in plants needs further investigation, and in this perspective, cell cultures appear a very useful tool.

## Figures and Tables

**Table 1:** Biotic programmed cell death (PCD) inducers in plant cell cultures.

Plant Species	PCD Induced by	Main Characteristics of Induced PCD	Reference
*Acer pseudoplatanus* L.	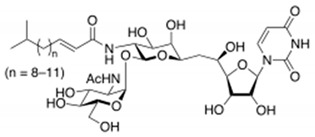 Tunicamycin	H_2_O_2_ accumulation, changes in cell and nucleus morphology, DNA fragmentation	[7]
*Acer pseudoplatanus* L.	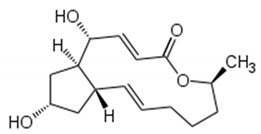 Brefeldin A	H_2_O_2_ accumulation, changes in cell and nucleus morphology, DNA fragmentation	[7]
*Acer pseudoplatanus* L.	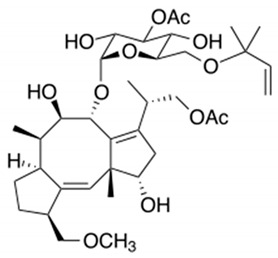 Fusicoccin	H_2_O_2_ accumulation, changes in cell and nucleus morphology, DNA laddering	[8]
*Arabidopsis thaliana* (L.) Heynh.	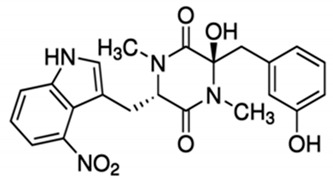 Thaxtomin A	Activation of gene transcription and protein synthesis, DNA fragmentation	[9]
*Nicotiana tabacum* L. cv. Bright Yellow 2	Metabolic products present in the *Alternaria alternata* culture filtrate	Cytoplasm shrinkage, chromatin condensation, DNA laddering	[10]
*Nicotiana tabacum* L. cv. NC89	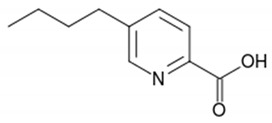 Fusaric acid	H_2_O_2_ accumulation, lipid peroxidation, caspase-3-like protease activity, mitochondrial dysfunction	[11]
*Nicotiana tabacum* L. cv. Bright Yellow 2	Culture filtrates of *Erwinia carotovora*	Changes in vacuole shape, endoplasmic actin filaments disassembly	[12]
*Nicotiana tabacum* L. cv. Bright Yellow 2	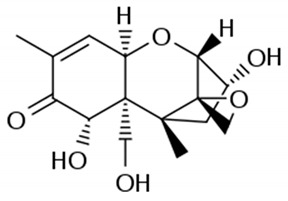 Deoxynivalenol	H_2_O_2_ accumulation, activation of gene transcription, mitochondrial dysfunction	[13]
*Arabidopsis thaliana* (L.) Heynh.	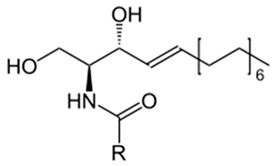 Ceramides	Generation of Ca^2+^ transient, H_2_O_2_ accumulation	[14]
*Solanum lycopersicum* L.	Phosphatidic acid	ROS and ethylene accumulation, protoplast shrinkage, nucleus condensation, caspase-3-like protease activity	[15]
*Nicotiana tabacum* L. cv. Bright Yellow 2	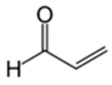 Acrolein	ROS accumulation, caspase-3-like protease activity	[16]
*Nicotiana tabacum* L. cv. Bright Yellow 2	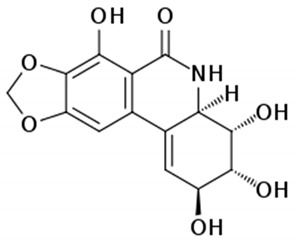 Narciclasin	Cell shrinkage, chromatin condensation, and nuclear DNA degradation	[17]
*Populus alba* L.	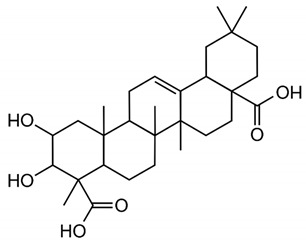 Medicagenic acid	ROS and RNS accumulation, changes in cell and nucleus morphology, chromatin condensation	[18]
*Nicotiana tabacum* L. cv. Bright Yellow 2	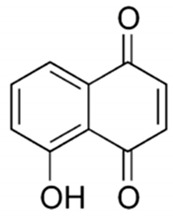 Juglone	ROS accumulation, DNA fragmentation and hypomethylation	[19]
*Vitis labrusca* L.	Alanine	DNA fragmentation, expression of defense-related genes, accumulation of phenolic compounds	[20]
*Arabidopsis thaliana* (L.) Heynh.	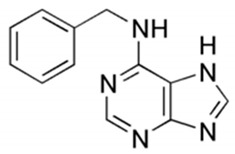 6-benzylaminopurine	Changes in nucleus morphology, DNA laddering, activation of gene transcription	[21]
*Arabidopsis thaliana* (L.) Heynh.	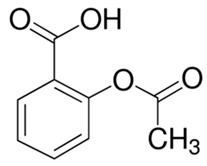 Acetylsalicilic acid	Cell shrinkage, nuclear DNA degradation, mitochondrial dysfunction, induction of caspase-like activity	[22]
*Acer pseudoplatanus* L.	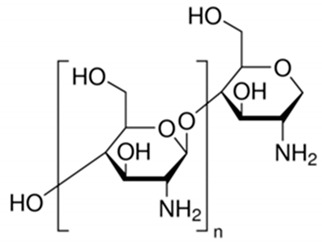 Chitosan	ROS and RNS accumulation, nuclear DNA degradation, caspase-3-like protease activity, mitochondrial dysfunction, expression of defense-related genes	[23]

**Table 2:** Abiotic PCD inducers in plant cell cultures.

Plant Species	PCD Induced by	Main Characteristics of Induced PCD	Reference
*Nicotiana tabacum* L. cv. Bright Yellow 2	Cadmium ions	Changes in cell and nucleus morphology, appearance of autophagic bodies	[31]
*Nicotiana tabacum* L. cv. Bright Yellow 2	Aluminium oxide nanoparticles	Caspase-like protease activity, mitochondrial dysfunction, DNA fragmentation	[32]
*Viola tricolor* L.	Zinc and lead ions	Changes in cell and nucleus morphology, DNA fragmentation, caspase-like and papain-like cysteine protease activity	[33]
*Nicotiana tabacum* L. cv. Bright Yellow 2	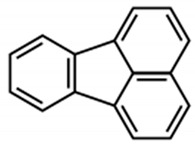 Fluoranthene	ROS accumulation, lipid peroxidation, caspase-3-like protease activity, DNA fragmentation	[34]
*Arabidopsis thaliana* (L.) Heynh.	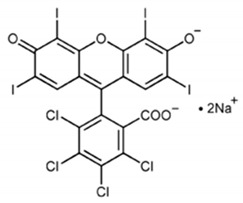 Rose Bengal	ROS accumulation, lipid peroxidation, specific gene activation	[35]
*Glycine max* (L.) Merr.	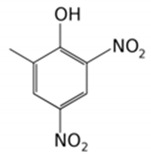 Dinitro-*o*-cresol	Mitochondrial dysfunction, DNA fragmentation, caspase-3-like protease activity	[36]
*Populus euphratica* Oliv.	1-Butanol	Cell shrinkage, chromatin condensation, nuclear DNA degradation, caspase-3-like protease activity	[37]
*Populus euphratica* Oliv.	ATP (externally added)	Elevation of cytosolic Ca^2+^ levels, ROS accumulation, mitochondrial dysfunction	[38]
*Nicotiana tabacum* L. cv. Bright Yellow 2	Heat stress	ROS accumulation, cytoplasmic shrinkage, DNA fragmentation, caspase-3-like protease activity, mitochondrial dysfunction, induction of defense-related genes	[39]
*Cakile maritime* Scop. *Arabidopsis thaliana* (L.) Heynh.	Salt stress	ROS accumulation, lipid peroxidation, specific gene activation, caspase-3-like protease activity, mitochondrial dysfunction	[40]
*Arabidopsis thaliana* (L.) Heynh.	Ozone	ROS accumulation, Ca^2+^ influx, changes in cell and nucleus morphology	[41]
*Vitis vinifera* L.	Darkness (in senescent cultures)	ROS accumulation, DNA fragmentation, caspase-3-like protease activity, mitochondrial dysfunction	[42]
*Nicotiana tabacum* L. cv. Bright Yellow 2	UV-B	ROS accumulation, DNA fragmentation, mitochondrial dysfunction	[43]
*Pinus pinaster* Ait.	Sugar and phosphate depletion	Changes in cell and nucleus morphology, DNA fragmentation, DNA laddering	[44]
*Arabidopsis thaliana* (L.) Heynh.	polyethylene glycol, mannose, H_2_O_2_, ethylene *	Changes in cell and nucleus morphology, DNA fragmentation, specific gene activation. * Cell plasma membrane permeabilization, ROS overproduction, severe oxidative stress	[45]

* Main characteristics of PCD induced by ethylene.

## Abbreviations

HS	Heat stress
PCD	Programmed cell death
RNS	Reactive nitrogen species
ROS	Reactive oxygen species
